# The treatment effects of Trametes Robiniophila Murr against colorectal cancer: A mini-review

**DOI:** 10.3389/fmed.2022.981516

**Published:** 2022-08-03

**Authors:** Bo Li, Qian Cao, Zhuo Liu

**Affiliations:** ^1^Department of Rehabilitation Medicine, China-Japan Union Hospital of Jilin University, Changchun, China; ^2^Department of Education, Jilin University Second Hospital, Changchun, China; ^3^Department of Gastrointestinal Colorectal and Anal Surgery, China-Japan Union Hospital of Jilin University, Changchun, China

**Keywords:** colorectal cancer, Trametes Robiniophila Murr, Huaier, mechanisms, anticancer

## Abstract

Colorectal cancer (CRC) is a worldwide disease threatening people's lives. Surgery and chemotherapy are still the main methods for CRC treatment. However, the side effects and chemotherapeutic drug resistance restrict the application of chemotherapy. Trametes Robiniophila Murr, also known as Huaier, is a traditional Chinese medicine that has been used for more than 1,600 years. Huaier extracts have promising anti-cancer effects on hepatoma, breast cancer, and gastric cancer. Nowadays, the tumor inhibition of Huaier on CRC has attracted more and more attention. This review mainly provides the possible anti-tumor mechanisms of Huaier for CRC treatment in apoptosis and inhibiting proliferation of tumor cells, preventing epithelial-mesenchymal transformation (EMT), weakening proliferation and differentiation of CRC stem cells, decreasing the vessel density in tumor tissues, and enhancing the immune system and chemotherapeutic efficacy. Huaier extract may be a good candidate for CRC treatment, especially when combined with other chemotherapeutic agents.

## Introduction

Colorectal cancer (CRC) is a common malignant tumor in the digestive tract, and its incidence is increasing. The mortality of CRC is on the rise and ranks third among all malignancies (1) that seriously endanger the health of people. Surgery is still the most important current treatment method for CRC, but the average 5-year survival rate is < 50%, and about 30% of patients may develop tumor recurrence after surgery (2). Chemotherapy and radiotherapy are still the most commonly used methods to prevent the post-surgical tumor recurrence or treat the advanced CRC that is unsuitable for surgical resection. However, the side effects of chemotherapy and radiotherapy cannot be restricted nowadays. Therefore, developing new anti-cancer drugs with low toxicity and drug resistance is urgent. More and more researchers are focusing on the effects of Chinese medicine in postoperative adjuvant therapy for many years. Post-surgical adjuvant therapy by Chinese medicine can effectively enhance the effect of chemotherapy, reduce the toxic side effects of chemotherapy and adverse reactions caused by surgery, etc., and improve the survival rate and the quality of life of CRC patients (3, 4).

**Figure 1:**
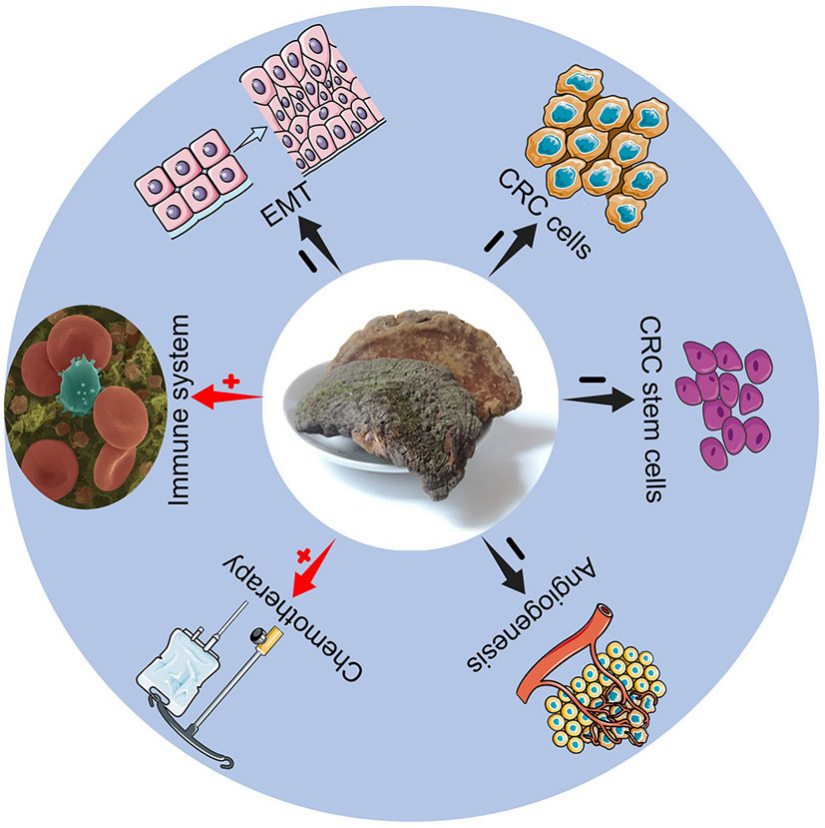
Mechanisms of Trametes Robiniophila Murr for CRC treatment.

Trametes Robiniophila Murr, also known as Huaier, has a history of more than 1,600 years as traditional Chinese medicine. Huaier granules are common clinical pharmaceutical agents. Huaier is a medicinal fungus that grows on acacia, locust tree, sandalwood and many other trees, and it contains various organic components and more than 10 minerals. The main ingredient of Huaier is the fungal matter, which includes polysaccharides, proteins, ketones, and alkaloids, and the active ingredient is polysaccharide-protein (5). Huaier granules have shown promising tumor inhibitory effects on many kinds of cancers, including hepatic cancer (6), breast cancer (7), and gastric cancer (8). It is reported that Huaier extracts can effectively inhibit the proliferation of colon cancer cells (9) and prevent the progression of colon tumors in nude mice (10). These findings may provide new aspects for CRC treatment. However, the concrete mechanisms of Huaier against CRC are not clear. As a result, this review mainly provides the possible anti-tumor mechanisms of Huaier for CRC treatment (Figure 1).

## The role of Huaier in CRC cells

### Promoting apoptosis and inhibiting proliferation of tumor cells

Huaier can inhibit the proliferation of CRC cells by inducing tumor apoptosis. It is reported that Huaier extract can induce G0/G1 and S phrases arrest, and the proliferation ability of tumor cells is weakened and the ability of apoptosis is enhanced (11, 12). Studies have shown that wild-type p53 protein encoded by the p53 gene inhibits cell growth, induces apoptosis, and repairs damaged cells. These functions are related to genomic stability, cell cycle progression, apoptosis, and DNA damage repair (13). Bcl-2 can regulate tumor cell apoptosis and inhibit cancer cell proliferation through the mitochondrial pathway (14). Lin *et al*. found that Huaier extract can improve the severity of inflammatory bowel disease-related tumors, induce apoptosis of related tumor cells, and inhibit tumor cell proliferation in CRC mouse model. In addition, the apoptosis-associated protein levels, including p53 and Bcl-2, show significant differences when treated with Huaier in CRC cell lines (15). Therefore, Huaier may possess good properties to induce apoptosis of CRC cells by activating and upregulating p53 and downregulating Bcl-2/Bax genes. Sun *et al*. have reported that Huaier granules can significantly decrease the tumor development in nude mice transplanted with HT-29 colon carcinoma cell line by downregulating the expression of PI3KR1, Akt, Wnt1, CTTNB1, and Notch genes (9). It is reported that chromosomal maintenance protein is a target for Huaier in hepatocellular carcinoma, and Huaier inhibits the cell cycle of liver cancer cells by regulating chromosomal maintenance proteins, thereby inhibiting tumors (11). In addition, Huaier extracts can inhibit proliferation and induction of apoptosis in two tuberous sclerosis complex cell models by inhibiting JAK2/STAT3 and MAPK signaling pathways (16). However, the above mechanisms of Huaier should be further investigated in CRC cell lines or animal models.

### Inhibiting epithelial-mesenchymal transformation

Epithelial-mesenchymal transformation (EMT) refers to the biological process by which epithelial cells are transformed into cells with interstitial phenotypes through specific procedures (17). EMT is always associated with tumor invasion and distant metastasis (18). Matrix metalloproteinase (MMP) plays an essential role in the EMT by influencing the degradation and remodeling of the extracellular matrix, increasing local invasion of tumor cells, and improving distant metastasis (19). Researchers found that Huaier can decrease the expressions of Bcl-2, MMP-2, and MMP-9 in the MKN-45 cell line, thus inducing apoptosis and preventing the invasion of tumor cells (20). Huaier may reduce the invasion and metastasis of CRC cells by inhibiting EMT. The effects may be associated with the regulation of the expression of messenger RNA and the transcription factors. Furthermore, Huaier extracts can slow the growth of pancreatic cancer and decrease the invasion, migration, and EMT of pancreatic cancer cells by suppressing Wnt/beta-catenin pathway (21). However, the inhibitory effect of Huaier on EMT in CRC treatment remains to be further studied.

## The role of Huaier in CRC stem cells

CRC stem cells have a stronger ability to proliferate, infiltrate, and metastasize than CRC cells (22). Inhibiting the proliferation, invasion, and migration of tumor stem cells can decrease the proportion of stem cells transformed into cancer cells, thus preventing the recurrence and migration of tumors (23). The proliferation, differentiation, and self-renew ability of CRC stem cells are regulated by multiple signaling pathways. The expression of messenger RNA associated with tumor proliferation in CRC stem cells treated with Huaier granules is significantly downregulated, and the proliferation of stem cells is inhibited (9). Huaier extracts inhibit the migration of CRC and may be achieved by down-regulating the expression of messenger RNA of key genes or proteins to attenuate the properties of cancer stem cells. Detailed mechanisms about the effect of Huaier on CRC stem cells need to be further verified.

## The role of Huaier in tumor angiogenesis

Neovascularization is a typical feature of tumors and is a necessary process for tumor invasion and distant metastasis (24, 25). Neogenesis in tumors is regulated by various angiogenesis factors, such as vascular endothelial growth factor (VEGF) and its regulatory gene hypoxia inducing factor 1α, human macrophage metal elastase (26, 27). The overexpression of VEGF is strongly associated with poor treatment outcomes and reduced survival rates in cancer patients (28, 29). Therefore, inhibiting the proliferation of vascular endothelial cells induced by VEGF is the key to preventing tumor invasion and migration. Zheng *et al*. found that Huaier polysaccharide (TP-1) can reduce the expression of hypoxia-inducible factor and VEGF in tumor tissues in mice bearing hepatocellular carcinoma SMMC-7721 tumors model (30). Huaier extracts can not only decrease VEGF levels in mouse mammary tumor cells but also decrease microvessel density in tumor tissues (31). Huaier granules can suppress the infiltration of tumor-associated macrophages, and inhibit the angiogenesis of macrophages, thereby inhibiting tumor progression in RAW264.7 murine macrophage cell line (32). However, this effect has not been tested in CRC.

Huaier extracts can inhibit the invasion and migration of CRC by inhibiting neovascularization in the tumor, which may be the focus of future clinical research and can be applied in the clinical treatment of CRC. However, more experiments are needed to validate and explore the possible molecular mechanisms of the effects of Huaier on tumor angiogenesis.

## The role of Huaier in the immune system

The balance between tumor-specific immunity and tolerance affects the health of the host. Under the protection of the immune system, a normal organism can prevent the deterioration of mutated tumor cells. However, cellular immune function in CRC gradually decreases and continues to deteriorate with tumor progress, recurrence, and distant metastasis (33, 34). Therefore, it is of great significance to protect and enhance the immune defense ability to increase the efficacy of anti-tumor therapy for CRC patients. As a traditional Chinese medicine, Huaier extracts can act as an effective immune enhancer and modulator by fully mobilizing cellular and humoral immunity, thus reducing the cachexia caused by chemotherapy (5, 35). Huaier granules can enhance the phagocytosis of macrophages, increase the number and activity of natural killer cells, and enhance the body's immunity to effectively induce the death of tumor cells (19, 36). Huaier granules can promote the maturation of dendritic cells, and the dendritic cells treated by Huaier can significantly stimulate the proliferation of CD4+T cells and promote their differentiation into the Th1 subgroup (37). In one study, the researchers found that Huaier polysaccharides can decrease the nephrotoxicity caused by cisplatin chemotherapy and protect renal function by regulating PI3K/Akt/mTOR signaling pathway *in vitro*, thus enhancing the immune ability of patients (38). Huaier extracts can perform the anti-tumor effects by enhancing the immune system, but detailed mechanisms still need further study.

## Enhancing chemotherapeutic efficacy

Clinically, the main causes of tumor deaths are metastasis and recurrence (39, 40). Tumor metastasis is a multistep process in which tumor cells penetrate stromal tissue, blood, or lymph node metastasis, adhere to the basement membrane, and invade the target organ (41, 42). Although chemotherapy, radiotherapy, and targeted therapy can decrease the recurrence and metastasis of cancer, there are still some problems that need to be solved, such as insensitivity to chemotherapy drugs and drug resistance. Chemotherapy still plays a dominant role in the comprehensive treatment of CRC, so it is significant to enhance chemotherapy sensitivity. The inhibition of EMT can increase the chemotherapy sensitivity of CRC to oxaliplatin (43). It is reported that Huaier extracts can prevent EMT (21), which may be used to increase chemotherapy sensitivity. Studies have shown that Huaier plays an effect on the reversal of chemotherapeutic agents resistance, thus increasing the chemotherapy effects (44, 45). The efficacy of Huaier combined with paclitaxel in treating BT474 and MDA-MB-231 breast cancer-bearing mice is superior to that of paclitaxel alone. The combination of Huaier and paclitaxel can reduce the levels of PI3K and p-AKT (46). In one meta-analysis, the researchers concluded that Huaier granules can enhance the chemotherapeutic efficacy of gastric cancer (8). Huaier granules can increase the sensitivity of chemotherapy and inhibit the recurrence and metastasis of tumors. However, a large number of basic and clinical studies need to confirm its effect on CRC and its specific mechanism.

## Conclusions and perspectives

There are still some problems that need to be solved to be widely applied in CRC treatment. (1) The active ingredient of Huaier for tumor treatment has not been determined. (2) Although increasing studies have focused on the direct effects of Huaier on cancer cells, few studies have explored its molecular and immunomodulatory mechanisms. (3) Whether the inhibitory effect of Huaier granules on the invasion and migration of CRC is related to the influence of intestinal flora needs further study. (4) More basic and clinical studies should be performed to provide a more convincing basis for applying Huaier in the treatment of CRC.

In conclusion, Huaier extracts may inhibit the progression of CRC in various ways, include inducing apoptosis and inhibition of tumor cell proliferation, blocking epithelial mesenchymal transition (EMT), attenuating proliferation and differentiation of CRC stem cells, reducing vascular density in tumor tissue, and enhancing the immune system. Therefore, Huaier may be an excellent candidate to enhance the sensitivity of chemotherapy while enhancing the immune system and decreasing side effects. The genes, signaling pathways, and related molecular mechanisms involved in the interaction between Huaier extracts and CRC are the focuses of future research.

## Author Contributions

BL drafted the review. QC generated the graph and guided the construction of the manuscript. ZL edited the review. All authors contributed to the article and approved the submitted version.

## Funding

This study was supported by Science and Technology Department of Jilin Province (20200201325JC).

## Conflict of interest

The authors declare that this study received funding from Qidong Gaitianli Pharmaceutical Co., LTD. The funder was not involved in the study design, collection, analysis, interpretation of data, the writing of this article or the decision to submit it for publication.

## Publisher's note

All claims expressed in this article are solely those of the authors and do not necessarily represent those of their affiliated organizations, or those of the publisher, the editors and the reviewers. Any product that may be evaluated in this article, or claim that may be made by its manufacturer, is not guaranteed or endorsed by the publisher.
